# Synergistic Adaptations of Yak Rumen Microbiota, Metabolites and Host to Altitudinal

**DOI:** 10.3390/microorganisms13071543

**Published:** 2025-06-30

**Authors:** Jianming Ren, Xiong Ma, Pengfei Zhao, Lan Zhang, Shiyu Tao, Xiangyan Wang, Bingang Shi

**Affiliations:** 1 College of Chemistry and Life Sciences, Gansu Normal University for Nationalities, Hezuo 747000, China; 0700085@gnun.edu.cn (J.R.); 0700090@gnun.edu.cn (P.Z.); 0700089@gnun.edu.cn (L.Z.); taosy1989@126.com (S.T.); shibg@gsau.edu.cn (B.S.); 2 College of Animal Science and Technology, Gansu Agricultural University, Lanzhou 730070, China; wxy9242022@163.com

**Keywords:** yak, rumen, microorganisms, metabolites

## Abstract

Rumen microbiota and metabolites play important roles in energy metabolism and immune regulation in the host. However, the underlying mechanisms of their interaction with the host to regulate yak plateau adaptation remain unknown. In this study, the effects of altitude on the rumen microbiome, metabolome, and fermentation parameters of yaks were analyzed. The fiber content of pasture grasses increased with altitude, while crude protein content was significantly higher at an altitude of 2800 m (T2800) compared to an altitude of 4500 m (T4500) (*p* < 0.05). The acetic acid, propionic acid, and volatile fatty acids of yaks in the T4500 group were significantly higher than in the T2800 group (*p* < 0.05). Simpson’s index of rumen microorganisms in the T4500 group of yaks was significantly higher than in T2800 and T3500 yaks. The relative abundance of *Rikenellaceae_RC9_gut_group* and *Succiniclasticum* was significantly higher in T4500 than in T2800, while *Prevotella* and *Streptococcus* were more abundant in T2800 than in T4500. Rumen metabolomics analyses revealed that yak rumen metabolites at different altitudes were influenced by forage and altitude, mainly affecting energy metabolism and fatty acid biosynthesis (such as purine and glycerophospholipid metabolism). In summary, altitude may influence rumen microbes and metabolites through pasture nutrient composition.

## 1. Introduction

The Tibetan Plateau has an ecological environment characterized by low oxygen levels, cold, and nutrient-deprived pastures [1,2]. Animals in high-altitude environments suffer from inadequate oxygen supply, impairing body function and physiology. Moreover, cold temperatures create a harsh environment, with temperatures dropping by about 6 °C for every kilometer of elevation gain. Additional factors include late winter and early spring snow cover, scarcity of pasture, and resultant weight loss [3]. To adapt to extreme terrestrial environments, species have developed specific characteristics through natural selection.

Yaks (*Bos grunniens*) live in the plateau area 3000 m above sea level, known as the “boat of the plateau,” and serve as an important economic source for local herdsmen by providing meat, milk, wool, and other resources [4]. Yaks have undergone natural and artificial selection over long periods, leading to breeds with different morphological, physiological, and adaptive characteristics, enhancing their survival in harsh environments [5]. Yaks have inhabited the Tibetan Plateau for a long time and are highly adaptable to high temperatures and low oxygen levels [3]. Reports indicate that yaks have specialized molecular mechanisms for adapting to cold and low-oxygen environments [6]. Altitude is a significant external environmental stressor affecting yak performance. Rumen microbiota and their metabolite-host interactions play a crucial role in yak acclimatization to high-altitude environments [7]. Previous studies on plateau adaptation have primarily focused on genetic variation, but growing evidence demonstrates that rumen microorganisms, as the “second genome” of the host, play an indispensable role in plateau acclimatization [8]. Numerous studies have shown that microbial communities can improve host development, disease resistance, and environmental adaptation [9]. There is increasing interest in studying rumen and gastrointestinal microbial adaptations to the environment. Studies have shown that changes in environment and forage can alter microbial communities and abundance, affecting their function [10].

The rumen, a complex micro-ecosystem, contains various microorganisms such as fungi and bacteria, which play key roles in ruminants [11]. These microorganisms decompose and synthesize food ingested by ruminants into the rumen, producing volatile fatty acids that are absorbed by the host and provide energy for the animal [12]. Ruminal microorganisms also have immunomodulatory and food fermentation functions in the host [13]. Changes in the microbial populations within the rumen result in corresponding changes in their physiological roles [10]. Current studies on rumen microbial metabolism primarily focus on metagenomics [14] and transcriptomics [15], and increasing research is examining rumen microbes and their metabolic functions. Microbial diversity is closely linked to the metabolic capacity and stability of the host organism [15]. Studies have shown that greater human gut flora diversity improves meal fermentation efficiency and contributes to intestinal ecosystem stability. Additionally, increased microbial diversity in the human gut reflects better health and stronger metabolism [16].

Previous research has shown the effects of diet, health, and age on rumen microorganisms. Yaks are differentially adapted to various altitudes. This study analyzed yak adaptation to altitude from a microbial perspective by exploring the potential effect of altitude on rumen microbiota and metabolism through 16S rRNA sequencing and metabolomics.

## 2. Materials and Methods

The animal study was approved by the Ethics Committee of Gansu Normal University for Nationalities (approval number GNUN-EC-2024-023).

### 2.1. Experimental Sample Collection

**Figure 1:**
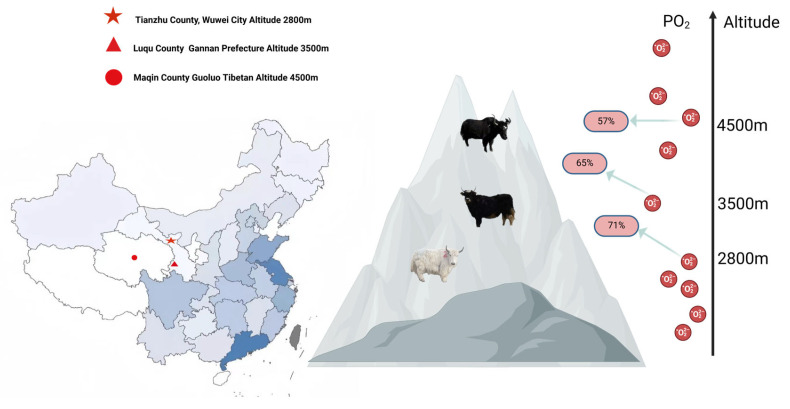
Collection sites of rumen fluid samples from yaks at three altitudes.

This study was conducted from 4 December 2024 to 27 December 2024 (dry grass period). Sampling was conducted in Maqin County, Guoluo Tibetan Autonomous Prefecture, Qinghai Province (elevation 4500 m, average temperatures of −8 °C to −10 °C); Larenguan Township, Luqu County, Gannan Tibetan Autonomous Prefecture, Gansu Province (elevation 3500 m, average temperatures of −5 °C to −13 °C); and Heimajuanhe Village, Songshan Township, Tianzhu Tibetan Autonomous County, Gansu Province (elevation 2800 m, average temperatures of −5 °C to −13 °C) (Figure 1). Selection of healthy male yaks around 5 years of age, with a grazing duration standardized at 8 h per day, from 7:00 AM to 3:00 PM for all animals, with free access to natural pasture during that time. Rumen fluid samples were extracted from six randomly selected healthy yaks of similar body weight from the local pastures. The detailed procedure is as follows: the entire rumen was collected, and the ruminal contents were scooped up using a sterile scalpel, filtered through four layers of sterile gauze, and divided into two 50 mL sterile cryogenic tubes. The tubes were labeled and immediately stored in liquid nitrogen for subsequent 16S rRNA gene analysis and determination of VFAs. The samples were then injected into liquid nitrogen for sequencing. To determine the nutrient composition of pasture grasses at different altitudes, 10 m × 10 m sample plots were set up in randomly selected pastures with uniformly distributed vegetation, flat terrain, and abundant plant taxa. Five sample squares, each measuring 0.5 m × 0.5 m, were selected diagonally within the plots. The samples were cut flush with the ground using scissors, weighed, and recorded. Inedible parts such as stones, soil, animal feces, and poisonous weeds were removed. The samples were air-dried, weighed, recorded, crushed, passed through a 1 mm sieve, and stored at room temperature for testing.

### 2.2. Determination of Forage Nutrient Content

Dry matter (DM), crude protein (CP), crude fat (EE), calcium (Ca), and phosphorus (P) in forages were analyzed according to the AOAC method [17]. The contents of acid detergent fiber (ADF) and neutral detergent fiber (NDF) were determined using the Van Soest method [18].

### 2.3. Determination of Rumen Fermentation Parameters

The pH of rumen fluid was measured using a portable pH meter (206-pH 1; Testo, RO) after extraction. The rumen fluid was thawed and centrifuged at 20,000× *g* and 4 °C for 15 min. Volatile fatty acids (VFAs) were determined by gas chromatography equipped with a megabore HP-MOLSIV column (Agilent Technologies, Palo Alto, CA, USA), following Wang et al. [19].

### 2.4. Metabolome Sequencing and Bioinformatics Analysis

Frozen rumen contents were thawed on ice, and 500 µL of 70% methanol internal standard extract was added. The mixture was shaken for 3 min and then left to stand for 30 min at −20 °C. It was then centrifuged for 10 min at 12,000 r/min. Next, 50 µL of the supernatant was aspirated and centrifuged again at 12,000 r/min for 5 min. Finally, 150 µL of the supernatant was placed in injection vials for data collection and further analysis [20]. The metabolome of rumen contents was analyzed using ultra-performance liquid chromatography (UPLC, Thermo Fisher Scientific, Waltham, MA, USA) and tandem mass spectrometry (MS/MS). An amount of 3 μL of the sample was separated using an HSS T3 chromatographic column (100 mm × 2.1 mm i.d., 1.8 μm) and then analyzed by mass spectrometry. Mobile phase A was 95% water + 5% acetonitrile (containing 0.1% formic acid), and mobile phase B was 47.5% acetonitrile + 47.5% isopropanol + 5% water (containing 0.1% formic acid). The flow rate was 0.40 mL/min, and the column temperature was 40 °C. Sample mass spectrometry signal acquisition uses a positive and negative ion scanning mode, with a mass scanning range of 70–1050 *m*/*z*. The sheath gas flow rate is 50 psi, the auxiliary gas flow rate is 13 psi, the auxiliary gas heating temperature is 425 °C, the positive mode ion spray voltage is set to 3500 V, the negative mode ion spray voltage is set to −3500 V, ion transfer tube temperature is 325 °C. Normalized collision energy is 20−40−60 V cyclic collision energy. First-stage mass spectrometry resolution is 60,000, second-stage mass spectrometry resolution is 7500, and data were collected using DDA mode. Peaks with less than 50% detection in QC samples were removed. After data quality control and preprocessing of metabolomics raw data, differential metabolites were screened and identified, and KEGG annotation of differential metabolites was performed.

### 2.5. Microbial Sequencing and Bioinformatics Analysis

Rumen microbial genomic DNA was extracted using a commercial kit. The quality of the extracted DNA was examined by 1% agarose electrophoresis. DNA was used as a template for 16S rRNA gene PCR amplification, using forward primer 27F (5′-AGAGTTTGATCCTGTGCTCAG-3′) and reverse primer 1492R (5′-TACGGCTACCTTTGTTACGACTT-3′). PCR products were quantified by the QuantiFluor™-ST Blue Fluorescence Quantification System (Promega, Madison, WI, USA) for library construction. Paired-end sequencing was performed following standard protocols using the Illumina HiSeq 2500 PE 250 System (Illumina, San Diego, CA, USA). Taxonomy was annotated using SILVA (version 138, https://www.arb-silva.de/). Sequencing data were analyzed using the QIIME (Quantitative Insights Into Microbial Ecology, version 1.9.1) pipeline. High-quality sequences were clustered into operational taxonomic units (OTUs) with 97% identity using UCLUST software (version 7.11). Weighted UniFrac distance-based principal coordinate analysis (PCoA) plots were constructed to illustrate significant differences between samples using R software (version 2.15.3). OTU data were statistically analyzed using the Kruskal–Wallis H-test method to determine differences between groups. The Linear Discriminant Analysis Effect Size (LEfSe) method was used to assess differences in microbial communities, with an LDA score threshold of 3.

### 2.6. Statistical Analysis

Forage nutrient composition was analyzed in SPSS software by incorporating a linear mixed-effects model, where “site” was treated as a random effect and “altitude” as a fixed effect. Rumen fermentation parameters were analyzed using SPSS software (version 26.0) through one-way analysis. Metabolomic statistics were analyzed based on retention time and ion current intensity, and the relative amount of each compound was calculated using MultiQuant software (version 2.0.2 by AB Sciex). Spearman’s correlation test was used to analyze correlations between rumen microorganisms, rumen metabolites, and rumen enzyme activities. Metabolomics data were subjected to univariate statistical analysis (including Student’s *t*-test) and multivariate statistical analysis (including principal component analysis (PCA)). The Metastats test in mothur (version 1.30.1) was used to identify rumen bacteria with differing abundances between groups. Significance was set at *p* < 0.05.

## 3. Results

### 3.1. Forage Nutrient Composition

The nutrient composition of pasture grasses at the three elevations is shown in Table 1. Ash increased with altitude (*p* < 0.05). CP in T2800 forage was higher than in T4500 (*p* < 0.05). ADF in T3500 forage was lower than in T2800 and T4500 (*p* < 0.05). NDF content gradually increased with altitude (*p* > 0.05).

### 3.2. Rumen Fermentation Parameters

The rumen fermentation parameters in yaks at different altitudes are shown in Table 2. Yak rumen total volatile fatty acids (TVFAs) were significantly lower in T2800 than in T3500 and T4500 (*p* < 0.05). Acetic acid and propionic acid contents were significantly lower in the T2800 group than in the T3500 and T4500 groups (*p* < 0.05). Isobutyrate content and pH were significantly higher in T2800 and T4500 than in T3500 (*p* < 0.05). The content of isovalerate in T4500 was significantly higher than in T2800 (*p* < 0.05). The percentage of A/P was significantly higher in T2800 (*p* < 0.05).

### 3.3. Analysis of Rumen Microbial Composition at Different Altitudes

**Figure 2:**
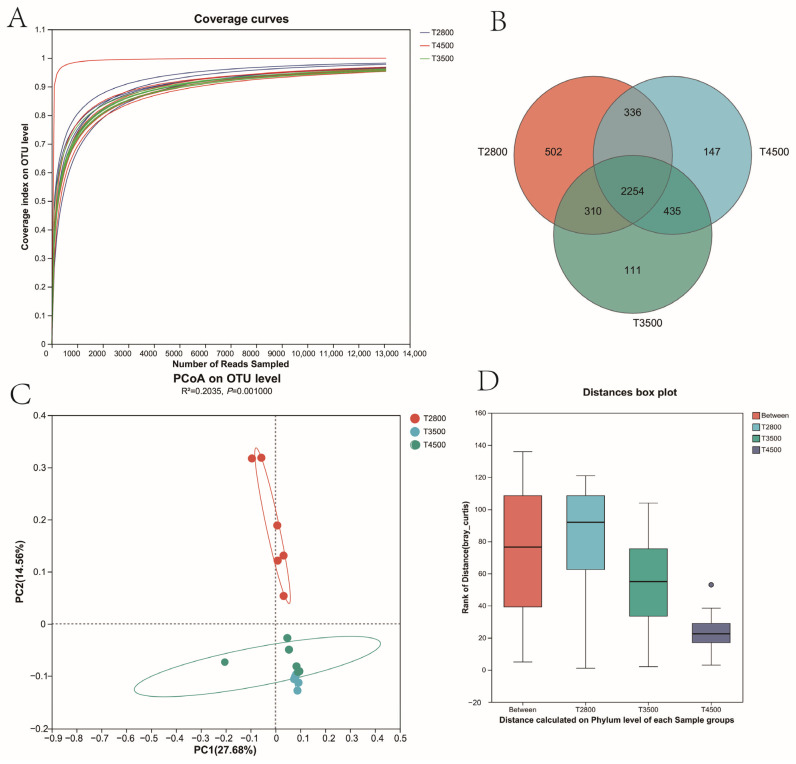
Diversity of rumen microbiota in yaks at different altitudes. (**A**) Dilution curve; (**B**) OTUs; (**C**) PCoA analysis; (**D**) Anosim analysis.

A total of 665,389 valid reads with an average length of 1452 bp were obtained from 18 groups of rumen fluid after screening and optimization. A total of 4031 OTUs were obtained by OTU clustering of non-repetitive sequences at 97% similarity after removing chimeras. Among these, 2919 OTUs were shared by the three groups, while 524, 155, and 266 OTUs were unique to T2800, T3500, and T4500, respectively (Figure 2B). Dilution curves were generated using observed OTUs of rumen microorganisms at each elevation to assess sampling depth for rumen bacterial composition (Figure 2A). The dilution curve stabilized around 3000, indicating saturation of sequencing coverage. Principal coordinate analysis (PCoA) showed that microbial diversity was similar in the T3500 and T4500 groups, while T2800 samples tended to separate from T3500 and T4500. This suggested that T2800 may harbor different microorganisms (Figure 2C). Furthermore, ANOSIM analysis showed that between-group differences were significantly greater than within-group differences (Figure 2D).

**Figure 3:**
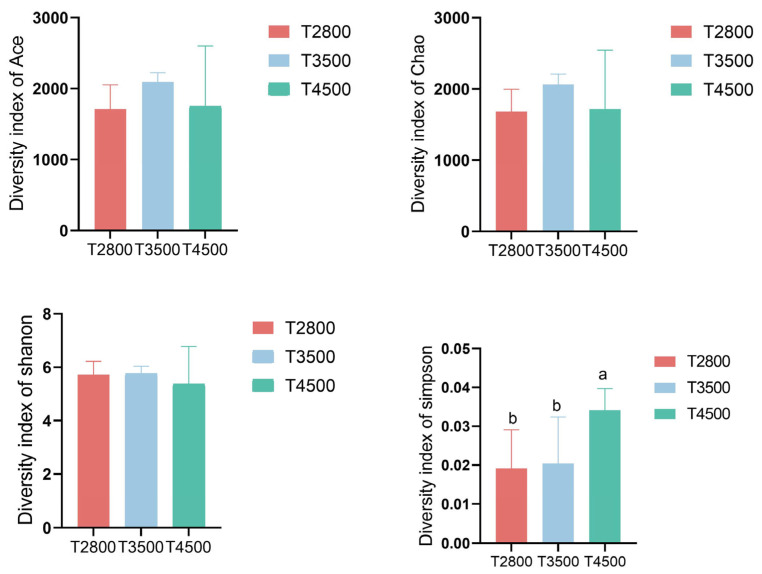
Microbial diversity indices for the rumen microbiota of yaks at different altitudes. Note: a b on the histogram indicates a significant difference.

The α-diversity ACE index and Chao index were significantly higher in the T3500 group than in the T2800 group, indicating greater microbial richness in T3500. However, the α-diversity Simpson index and Shannon index showed no significant differences among the three groups (Figure 3).

**Figure 4:**
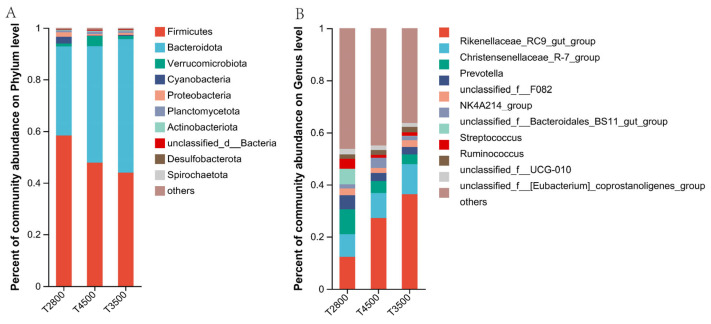
Microbial composition of the rumen microbiota in yaks at different altitudes. (**A**) Phylum; (**B**) genus.

A total of 26 phylum-level microorganisms were identified. Figure 4 presents the top 16 rumen microorganisms in relative abundance at the three elevations, with Firmicutes and Bacteroidota being the two main groups accounting for at least 92% of total microbial abundance at each elevation. A total of 325 genus-level microorganisms were identified in the three groups of rumen microorganisms in this study. Figure 4B illustrates the top 21 rumen microorganisms with the highest relative abundance.

The results showed that Firmicutes had the lowest relative abundance and Bacteroidota had the highest relative abundance in the T2800 group. Additionally, the proportions of Firmicutes and Bacteroidota were significantly higher in T2800 than in T3500 and T4500. A total of four phylum-level differential microorganisms were found among the microbiota of the three altitude groups. The relative abundance of unclassified_d__Bacteria, Bacteria, and WPS-2 increased with altitude, and the relative abundance in T4500 was significantly higher than in T2800. Conversely, the relative abundance of Elusimicrobiota in T2800 was higher than in T3500 and T4500 (Table 3).

The relative abundance of *Rikenellaceae_RC9_gut_group* and *Succiniclasticum* was significantly higher in T4500 than in T2800, but the relative abundance of *Prevotella* and *Streptococcus* was significantly higher in T2800 than in T4500. The relative abundance of *Papillibacter* was significantly greater in T3500 and T4500 than in T2800 (Table 4).

**Figure 5:**
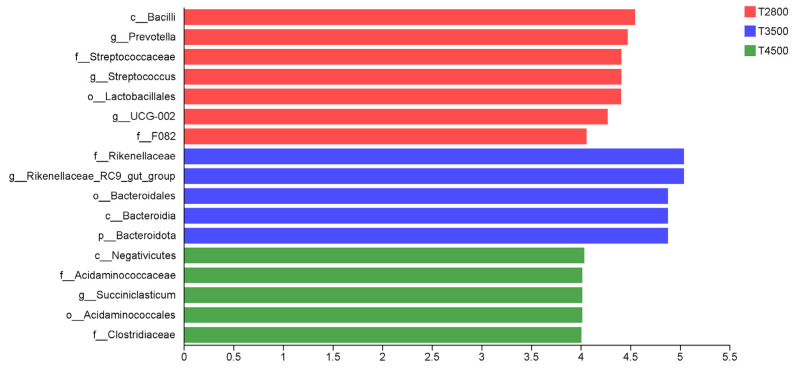
LEfSe histogram analysis. The bar graph length represents the effect sizes of the different species.

LEfSe analysis showed that *Prevotella*, *Streptococcus*, *Lactobacillales*, and *UCG-002* were significantly enriched in the T2800 group. *Rikenellaceae_RC9_gut_group* and *Bacteriodiota* were significantly enriched in T3500, while *Succiniclasticum* was significantly enriched in T4500. Thus, *Prevotella*, *Streptococcus*, *Lactobacillales*, and *UCG-002* can be potential biomarkers in T2800, and *Succiniclasticum* in T4500 (Figure 5).

**Figure 6:**
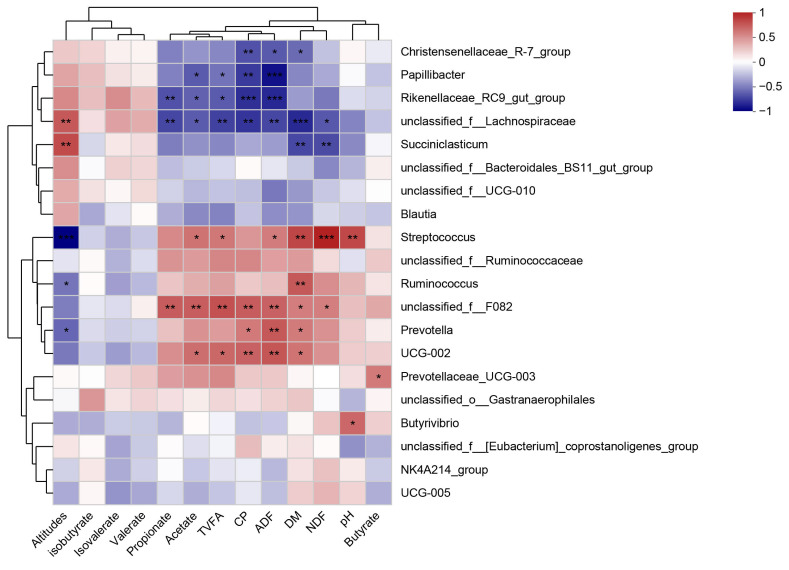
Correlation analysis of differential microorganisms with rumen fermentation parameters and forage nutrient content. Note: * indicates *p* < 0.05, ** indicates *p* < 0.01, *** indicates *p* < 0.001.

We performed correlation analyses of the top 20 microorganisms in abundance to screen for microorganisms associated with rumen fermentation parameters, elevation, and forage nutrients (Figure 6). The results indicated that *unclassified__f__Lachnospiraceae* and *Succiniclasticum* were positively correlated with elevation. *Unclassified__f__F082* was positively correlated with propionic acid content, while *Rikenellaceae_RC9_gut_group* and *unclassified__f__Lachnospiraceae* were negatively correlated with propionic acid content. *Papillibacter* and *unclassified__f__Lachnospiraceae* were negatively correlated with acetic acid content, while *Streptococcus* and *unclassified__f__F082* were negatively correlated with acetic acid content. Notably, *Prevotella* was positively correlated with forage dry matter content and CP, while *Rikenellaceae_RC9_gut_group* was negatively correlated with them; this was based on the association with microorganisms and forage nutrients. *Papillibacter*, *Rikenellaceae_RC9_gut_group*, and *unclassified__f__Lachnospiraceae* were negatively correlated with TVFAs content. In contrast, *Streptococcus* and *unclassified__f__F082* were moderately positively correlated with TVFAs.

### 3.4. Characterization of Rumen Metabolites in Yaks at Different Altitudes

**Figure 7:**
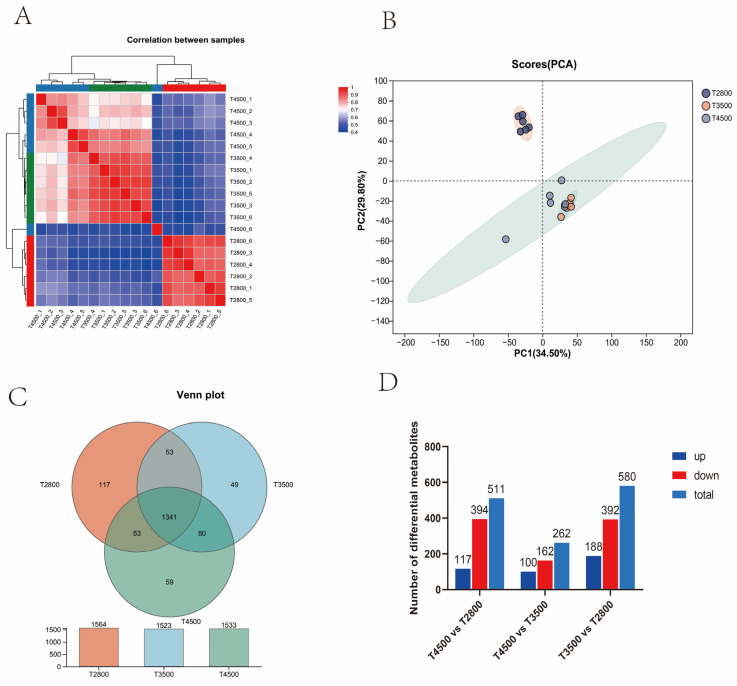
(**A**) Rumen metabolomics PCA of the three groups of samples; (**B**) Venn diagram; (**C**) Sample correlation; and (**D**) Comparison group histogram.

A total of 1866 metabolites were identified in 18 rumen fluid samples. PLS-DA score plots showed good clustering with significant intergroup differences. The association with the 18 rumen fluid samples was good, as shown in Figure 7A. According to the PCA results (Figure 7B), there was a clear separation of metabolites between the T2800, T3500, and T4500 groups, but T3500 and T4500 were clustered together, suggesting that different rumen metabolites existed in T2800, T3500, and T4500. There were a total of 1341 metabolites in the three groups, with 117, 49, and 59 unique metabolites in T2800, T3500, and T4500, respectively (Figure 7C). Differential metabolites were screened between the two groups using the *p* < 0.05 and VIP > 1 criteria. A total of 580 differential metabolites were identified between T2800 and T3500; 188 were up-regulated, and 392 were down-regulated. In T4500 vs. T2800, 511 differential metabolites were identified; 117 were up-regulated, and 394 were down-regulated. For T4500 vs. T3500, 262 differential metabolites were identified; 100 were up-regulated, and 162 were down-regulated (Figure 7D).

**Figure 8:**
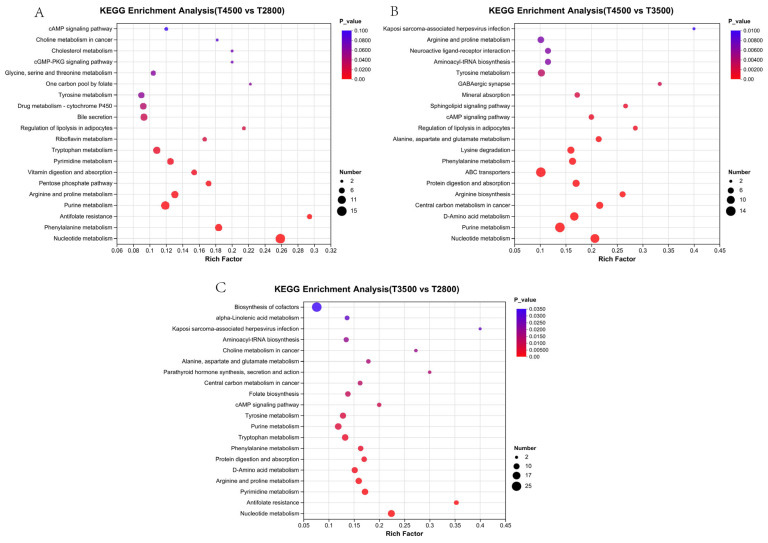
Differential metabolite enrichment pathways analysis for comparisons. (**A**) T4500 vs. T2800; (**B**) T4500 vs. T3500; (**C**) T3500 vs. T2800.

Further investigation using KEGG functional annotation and topological analysis of differential metabolites showed that rumen differential metabolites were mainly enriched in nucleotide and amino acid metabolism-related pathways, such as purine metabolism and arginine and proline metabolism at the three altitudes. T4500 vs. T2800 differential metabolites were mainly enriched in the cAMP signaling pathway, cholesterol metabolism, and cGMP-PKG signaling pathway (Figure 8A). T4500 vs. T3500 differential metabolites were mainly enriched in arginine and proline metabolism, tyrosine metabolism, and the sphingolipid signaling pathway (Figure 8B). T3500 vs. T2800 differential metabolites were mainly enriched in the biosynthesis of cofactors, alpha-linolenic acid metabolism, and alanine, aspartate, and glutamate metabolism (Figure 8C).

**Figure 9:**
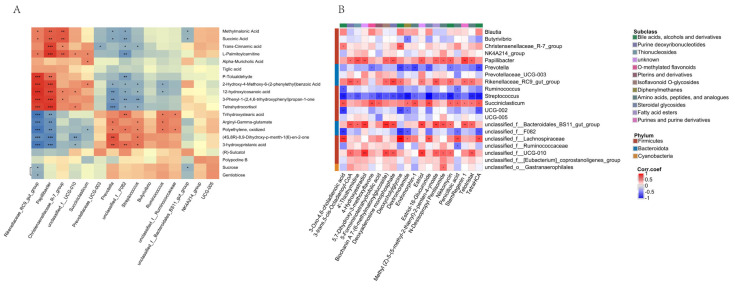
(**A**) The differential metabolite-microbe correlations; (**B**) Microbial correlates of metabolites with increasing altitude. Note: (**A**): The horizontal axis represents microorganisms, and the vertical axis represents metabolites. (**B**): The horizontal axis represents metabolites, and the vertical axis represents microorganisms. Note: * indicates *p* < 0.05, ** indicates *p* < 0.01, *** indicates *p* < 0.001.

Spearman correlation coefficient modeling was used to analyze the relationship between genus-level microorganisms and differential metabolites (*p* < 0.05 and R > 0.60). Significant associations were found between *Rikenellaceae_RC9_gut_group* and methylmalonic acid, succinic acid, and L-palmitoylcarnitine, with a significant positive association, and *Rikenellaceae_RC9_gut_group* showed a negative association with trihydroxystearic acid, arginyl-gamma-glutamate, and oxidized polyethylene. *Succiniclasticum* showed a significant positive association with L-palmitoylcarnitine and alpha-muricholic acid (Figure 9A). In addition, we analyzed the association between the metabolites that increased with altitude and the top 20 microorganisms in abundance. We found that *Papillibacter*, *Rikenellaceae_RC9_gut_group*, and *Succiniclasticum, unclassified f-Bacteroidales-BS11-gut-group*, and *unclassified f-Lachnospiraceae* were positively correlated with metabolites such as 3-Oxo-4,6-choladienoic acid and Dextromorphin-1, while *Streptococcus* and *undefined_f_F082* associated negative correlated with metabolites such as 3-Oxo-4,6-choladienoic acid, Dextromorphin-1, and other metabolites (Figure 9B).

## 4. Discussion

The rumen contains numerous microbiota, such as bacteria, archaea, and fungi, known as “natural fermenters”. These microbes help the host digest cellulose and other complex carbohydrates, converting them into volatile fatty acids and microbial proteins, which provide energy to the host [21]. Rumen microbes also play an important role in host immunity [22], disease resistance and prevention [23], and energy homeostasis [24]. Ruminal microbes adapt to extreme plateau environments by digesting dietary carbohydrates and converting them to fatty acids. These fatty acids are absorbed through the rumen epithelium, serving as a major energy source for ruminants and influencing the expression of host genes related to energy metabolism. Thus, ruminal microbes co-evolve with the genome to adapt to extreme plateau environments [25]. Sha et al. found that Tibetan sheep rumen microorganisms synergistically regulate rumen fermentation and epithelial immune barrier function, thereby enhancing metabolism and immunity in plateau Tibetan sheep [26]. Few studies have examined the interaction between yak rumen microbiota and their metabolites at different altitudes in response to environmental conditions. Based on this, the present study compared rumen microorganisms, metabolites, and rumen fermentation parameters in yaks at three altitudes to investigate how yak rumen microorganisms assist yaks in adapting to the extreme plateau environment.

### 4.1. Effect of Altitude on Rumen Microorganisms of Yak

Ruminal pH affects microbial growth and is influenced by ruminal VFAs, ammonia, and salivary secretions. Ruminants rely on VFAs produced by rumen fermentation for 70% of their energy source [27]. Fiber undergoes rumen fermentation to produce mainly acetic and butyric acids, while propionic acid is derived from the fermentation of proteins, sugars, and starches. The different fatty acids produced by rumen microorganisms have different functions. Acetic acid is one of the important substrates for the synthesis of fatty acids in animal organisms, while propionic acid is mainly used as an important raw material for gluconeogenesis. Butyric acid is absorbed and converted into β-hydroxybutyric acid, which provides energy for muscle tissue and can also be converted into fatty acids through other pathways [28]. Since diet is one of the most critical factors affecting volatile fatty acids [29]. In this study, the highest VFAs and propionic acid levels were found in the rumen of yaks in the T4500 group, likely due to the higher crude fiber content of the pasture in T4500 than in the other two altitudes. The 2800 m region is characterized by mild temperatures and high crude protein content in the pasture grass. At 3500 m, the climate and plant community structure undergo noticeable changes. At 4500 m, the region faces extreme environmental challenges, including hypoxia, low temperatures, and nutrient-poor pasture grass. As altitude increases, the carbohydrate content of pasture grass decreases, while fiber content increases, leading to more efficient fermentation of structural carbohydrates in the diet by fiber-degrading microorganisms in the rumen, resulting in higher levels of volatile fatty acids. Han et al. found that Qinghai-Tibetan yaks living at mid-altitude had higher total acid and propionic acid content [30], and Zhang et al. found that ruminants living at high altitude had higher VFAs content, with significantly higher expression of VFA-related genes responsible for absorption in the rumen of cattle [10]. These findings are consistent with our results.

In summary, differences in altitude may lead to variations in temperature and precipitation, which in turn affect the nutritional quality of pasture grass. The pasture grass consumed by yaks subsequently influences the production of volatile fatty acids through rumen fermentation. There is an intrinsic confounding relationship between altitude, geographical location, and forage type in this study. Each altitude is represented by an independent site with unique environmental conditions and forage composition. Additionally, because of the seasonal nature of yak reproduction and physiology, yaks on the Qinghai-Tibet Plateau are typically slaughtered during the cold season. The dry season offers relatively stable nutritional and environmental conditions, enabling us to focus on the effects of altitude while minimizing seasonal confounding factors.

Yaks, a unique livestock species of the Qinghai-Tibet Plateau, are significantly affected by the cold, hypoxic environment, exhibiting clear seasonal characteristics. Slaughter typically occurs during the cold season. The dry season provides relatively stable nutritional and environmental conditions, allowing us to focus on studying the effects of altitude while minimizing seasonal influences.

### 4.2. Effect of Altitude on Microbial Diversity in the Rumen of Yak

The gastrointestinal microbiota plays an important role in host digestion and nutrient synthesis. Gut microbiota and its function may be closely linked to the adaptive capacity of high-altitude hosts [31]. Microbial genes in the rumen of high-altitude ruminants are enriched in the pathway for VFA production, providing persistent energy to the host. Additionally, the microbiome co-evolves with the host genome to adapt to extreme environments [10]. Current studies on adaptation in plateau animals focus on genomic analysis and tissue morphology but lack research on rumen microbes and metabolites. This study investigated the molecular mechanisms of rumen microbes in adapting to plateau environments by examining rumen microbes and rumen fluid metabolites in yaks at different altitudes. The results showed significant differences in rumen microbial abundance with altitude, highlighting the important role of rumen microbes in degrading forage cellulose and forming a mutualistic relationship with the host’s environment. Some rumen bacteria produce plant-degrading enzymes, such as cellulase, which degrade plant crude fiber into short-chain fatty acids that the host absorbs and utilizes [32]. Altitude influences the distribution, nutrient composition, and type of forage, affecting the diversity, composition, and function of rumen microbes [33]. Alpha diversity evaluates species multiplicity and diversity of environmental communities through statistical indices. The ACE and Chao1 indices assess the number of OTUs in samples, while the Shannon and Simpson indices synthesize species diversity in samples [34]. The Simpson indices indicated that microbial diversity was significantly higher in the T4500 group than in the T2800 and T3500 groups. This is consistent with the findings of W et al., who found that after controlling for the effects of body weight, diet, reproductive status, and origin, altitude differences affect the composition and diversity of the gut microbiota of wildhouse mice [35]. Li et al. found that altitude significantly affects the diet and gut microbiota of wild plateau pika [36]. Higher rumen microbial diversity represents a more stable rumen micro-ecosystem, important for maintaining health and energy balance in ruminants [37].

### 4.3. Effect of Altitude on the Microbial Composition of the Rumen of Yaks

To understand differences in rumen microorganisms in yaks at different altitudes, we analyzed the effect of altitude on rumen flora structure at the phylum and genus levels. In this study, Firmicutes and Bacteroidetes were the dominant phyla of rumen microorganisms in yaks at different altitudes, consistent with previous studies [38]. We found that the relative abundance of T4500 Bacteroidetes was significantly higher than that of T2800. Bacteroidetes provide nutrients to the body by degrading cellulose, pectin, and complex carbohydrates, which help improve the host’s nutrient utilization efficiency and immune function. The high Bacteroidetes in T4500 may be due to the higher crude fiber content in high-altitude pasture grasses, which influences the composition of fiber-degrading bacteria in the rumen microflora. Another important reason may be that the rumen of high-altitude yak contains abundant Bacteroides, which helps extract carbohydrates and other nutrients from harsh environments to meet their own energy needs and plays an important role in improving physical immunity and high-altitude adaptability. We also found that the Firmicutes/Bacteroidetes (F/B) ratio of rumen microorganisms was higher in T2800 yaks and was closely related to altitude [39]. This adaptation helps high-altitude yaks deposit more fat, providing energy and maintaining metabolic balance. Therefore, we hypothesized that the rumen microecology of yaks gradually adapts to low-altitude environments, leading to changes in microbial composition.

*Prevotella* includes many metabolically variable strains such as *Prevotellaceae_UCG-001* and *Prevotellaceae_UCG-003* that degrade rumen proteins, starch, peptides, hemicellulose, and pectin to VFAs and amino acids by secreted enzymes [40]. In this study, the abundance of *Prevotellaceae* was significantly higher in group T2800 than in T4500. This difference may relate to the nutrient composition of local pastures. The yaks in group T2800 were foraged on pasture with higher crude protein and dry matter content compared to group T4500. Therefore, the yaks in group T2800 required more *Prevotellaceae* to degrade proteins and starch in the pasture. *Rikenellaceae_RC9_gut_group* was the dominant genus, consistent with studies on grazing yaks [41] and cattle [42]. *Rikenellaceae_RC9_gut_group*, a member of the Rikenellaceae family, degrades cellulose and hemicellulose, producing acetate as the main end product [42]. This aligns with our findings, where both acetic acid and *Rikenellaceae_RC9_gut_group* were higher in T4500 than in T2800. This may be due to the high cellulose content in the T4500 pasture, which requires more *Rikenellaceae_RC9_gut_group* to degrade cellulose, producing more acetic acid in the rumen. The host absorbs this acetic acid through the rumen epithelium for energy. This process may contribute to the better adaptation of T4500 yaks to the plateau environment.

### 4.4. Effect of Altitude on Microbial Metabolites in the Rumen of Yak

Metabolites in the rumen are mainly absorbed through the rumen epithelium to perform functions. In this study, T4500 vs. T2800 yak rumen differential metabolites were enriched in pathways such as the degradation of valine, leucine, and isoleucine, and the biosynthesis of fatty acids. These pathways may help animals at high altitudes cope with cold stress and increase carbohydrate dependence, requiring more energy [43]. Another reason is the higher crude protein and crude fat content of pasture grasses at lower altitudes. Succiniclasticum utilizes succinate to produce propionate [44], which is associated with increased NDF content. This aligns with our findings that the abundance of Succiniclasticum in T4500 was significantly higher than in T2800. This may be because, with increasing altitude, the crude fiber and NDF content in forage increased, requiring more Succiniclasticum in the rumen to degrade NDF after feeding.

Rumen metabolites are transferred through the rumen epithelium to play functional roles. In this study, we screened rumen microorganisms and their associated pathways for altitude adaptation through joint analysis of rumen microorganisms and their metabolisms. Differential metabolites in T2800 vs. T4500 were enriched in pathways such as the cAMP signaling pathway, choline metabolism in cancer, and cholesterol metabolism. Notably, we also found pathways related to altitude acclimatization, such as arginine and proline metabolism. Microbiota can contribute to host resistance to low-temperature stress by stimulating arginine and proline metabolism [45]. Yak rumen microbes may enhance their adaptation to extreme plateau environments through arginine- and proline-related pathways and regulate intracellular sugar concentration by modulating hydroxyproline, influencing downstream pentose and glucuronide mutualism pathways. Similarly, we found the pentose phosphate pathway in T2800 and T4500. We hypothesized that yaks in the T4500 group adapted to their environment by stimulating arginine and proline pathways in the rumen to modulate the downstream pentose phosphate pathway when adapting to colder temperatures. This is consistent with the findings of other studies, such as those by Qiu et al., who found that yaks from high-altitude regions have a higher abundance of energy-related gene families compared to those from low-altitude regions [5]. Compared with other plains mammals, the Tibetan antelope genome shows signs of adaptive evolution and gene family expansion in genes related to energy metabolism and oxygen transport [46]. The above research indicates that the harsh natural environment of high-altitude regions has enabled yaks to develop adaptive energy metabolism mechanisms, thereby helping ruminants adapt to their environment.

### 4.5. Correlation of Rumen Microorganisms, Metabolites, and Fermentation Products in Yak

**Figure 10:**
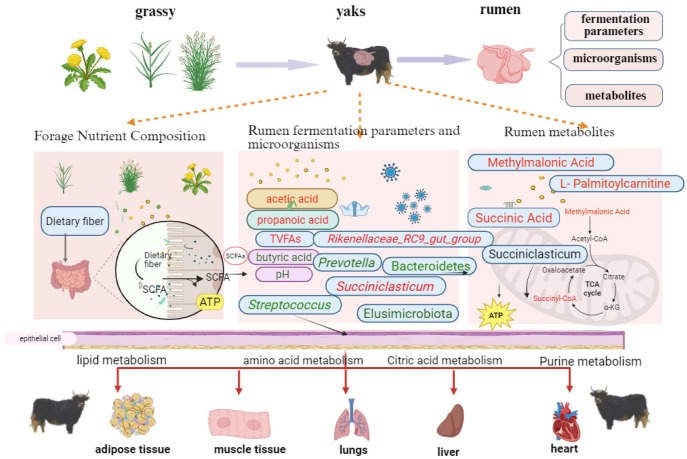
An interaction model of rumen fermentation parameters with their metabolites. Those in red font represent the increase in metabolites and microbial and rumen fermentation parameters with increasing altitude, while those in black font represent a decrease. Note: The red font indicates that the content increases with altitude, and the green font indicates that the content decreases with altitude.

We found that *Rikenellaceae_RC9_gut_group* and methylmalonic acid, succinic acid, and L-palmitoylcarnitine were significant factors by analyzing differential metabolites with microbial correlation. *Rikenellaceae_RC9_gut_group* primarily degrades cellulose and hemicellulose in the rumen [47,48]. Methylmalonic acid is related to the synthesis of malonyl coenzyme A, an important precursor of succinic acid in the TCA cycle [49]. We hypothesized that as altitude increases and temperature decreases, the cellulose content of local pasture gradually increases, requiring yaks to have a higher abundance of *Rikenellaceae_RC9_gut_group* in the rumen to degrade the cellulose and produce short-chain fatty acids. In the degradation of cellulose, energy-related precursors enter the bloodstream through the rumen epithelium and are absorbed by various tissues of the yak to provide energy (Figure 10). This adaptation may help yaks adjust to the plateau environment, but further experiments are needed to verify these reasons.

## 5. Conclusions

The analysis of forage nutrient composition and rumen fermentation parameters showed that crude fiber content increased and protein content decreased in forage at high altitudes. Total acid, propionic acid, and acetic acid content also increased with altitude. Additionally, rumen microbial diversity was higher in high-altitude yaks, and microorganisms associated with cellulose degradation became more abundant with elevation. These changes in microbial flora affected rumen metabolic profiles, leading to differences in purine and fatty acid metabolism, which may help yaks adapt to the plateau environment. Altitude influences the nutrient composition of local forage, such as crude fiber and crude protein content, and alters the rumen fermentation function of yaks. This, in turn, causes changes in yak rumen microorganisms and metabolites. During yak acclimatization to high altitudes, rumen microorganisms ferment the forage, producing more energy metabolism-related products. These metabolites are absorbed by the host, providing energy.

## Figures and Tables

**Table 1:** Nutrient composition analysis of pasture grasses at different elevations.

Group	Dry Matter DM (%)	Crude Protein CP (%)	Ash (%)	Neutral Detergent Fiber NDF (%)	Acid Detergent Fiber ADF (%)	Ether Extract EE (%)
T2800	94.58	6.27 ^a^	5.88 ^b^	64.53	26.00 ^b^	8.03 ^a^
T3500	93.73	4.67 ^c^	5.95 ^b^	67.60	20.33 ^c^	7.56 ^b^
T4500	93.21	5.77 ^b^	7.64 ^a^	69.00	31.11 ^a^	8.06 ^a^
SEM	0.06	0.87	0.88	0.14	0.05	0.31
*p*	0.555	0.001	0.001	0.535	0.001	0.001

Different lowercase letters in the same column indicated significant differences.

**Table 2:** Effect of altitude on rumen fermentation parameters in yak.

Fermentation Parameters	T2800	T3500	T4500	SEM	*p*-Value
pH	7.69 ^a^	7.64 ^a^	7.22 ^b^	0.01	0.032
NH_3_-N (mg/100 mL)	0.30	0.42	0.52	0.02	0.053
Acetic acid (%)	18.169 ^b^	20.54 ^b^	27.53 ^a^	1.33	0.039
Propionic acid (%)	4.76 ^b^	4.10 ^b^	7.98 ^a^	0.48	<0.001
Isobutyrate (%)	0.32 ^a^	0.16 ^b^	0.38 ^a^	0.18	0.037
Butyric acid (%)	3.13	2.96	3.04	0.08	0.965
Isovalerate (%)	0.31 ^b^	0.42 ^b^	1.19 ^a^	0.03	<0.001
Valerate (%)	0.52 ± 0.01 ^ab^	0.27 ± 0.04 ^b^	0.75 ± 0.01 ^a^	0.01	<0.001
A/P	4.61 ± 0.86 ^a^	5.05 ± 0.16 ^a^	3.46 ± 0.15 ^b^	0.29	0.042
TVFA (mmol/L)	28.27 ± 1.32 ^b^	28.47 ± 0.51 ^b^	39.65 ± 2.28 ^a^	0.90	0.045

Note: Superscripts a and b in the same row indicate a significant difference (*p* < 0.05) in different groups.

**Table 3:** Differential microorganisms at the phylum level.

Phylum	T2800	T3500	T4500	*p*	FDR
Bacteroidota	34.51 ± 12.85 ^b^	51.27 ± 7.27 ^a^	45.09 ± 21.55 ^a^	0.048	0.100
Fimicutes	58.33 ± 8.12 ^a^	43.97 ± 6.91 ^b^	40.97 ± 2.70 ^b^	0.004	0.047
p__unclassified_d__Bacteria	0.09 ± 0.07 ^b^	0.26 ± 0.12 ^a^	0.35 ± 0.22 ^a^	0.042	0.047
Campylobacterota	0.07 ± 0.08	0.005 ± 0.003	0.01 ± 0.004	0.025	0.100
WPS-2	0.00 ± 0.00 ^b^	0.03 ± 0.03 ^a^	0.06 ± 0.04 ^a^	0.008	0.047

Note: The superscript a b on the same row indicates a significant difference.

**Table 4:** Differential microorganisms at the genus level.

Genus	T2800	T3500	T4500	*p*	FDR
*Rikenellaceae_RC9_gut_group*	12.34 ± 6.40 ^b^	36.31 ± 6.78 ^a^	27.20 ± 14.17 ^a^	0.009	0.030
*Prevotella*	9.61 ± 5.46 ^a^	3.73 ± 1.35 ^b^	4.61 ± 3.83 ^b^	0.023	0.297
*Streptococcus*	6.02 ± 8.33 ^a^	0.13 ± 0.06 ^b^	0.04 ± 0.03 ^b^	0.001	0.201
*Succiniclasticum*	0.831 ± 0.16 ^b^	1.30 ± 0.66 ^b^	3.05 ± 3.34 ^a^	0.019	0.244
*Papillibacter*	0.59 ± 0.43 ^b^	1.67 ± 0.42 ^a^	1.12 ± 0.56 ^b^	0.011	0.087

Note: The superscript a b on the same row indicates a significant difference.

## Data Availability

The data provided in the study are stored in the Sequence Read Archive (SRA) repository under the accession number PRJNA 1166940.
